# Exogenous application of calcium to 24-epibrassinosteroid pre-treated tomato seedlings mitigates NaCl toxicity by modifying ascorbate–glutathione cycle and secondary metabolites

**DOI:** 10.1038/s41598-018-31917-1

**Published:** 2018-09-10

**Authors:** Parvaiz Ahmad, Elsayed Fathi Abd_Allah, Mohammed Nasser Alyemeni, Leonard Wijaya, Pravej Alam, Renu Bhardwaj, Kadambot H. M. Siddique

**Affiliations:** 1 Botany and Microbiology Department, College of Science, King Saud University, P.O. Box 2460, Riyadh, 11451 Saudi Arabia; 2 Department of Botany, S.P. College, Srinagar, 190001 Jammu and Kashmir India; 3 Plant Production Department, College of Food and Agricultural Sciences, King Saud University, Riyadh, Saudi Arabia; 4 Biology Department, College of Science and Humanities, Prince Sattam bin Abdulaziz University, 11942 Alkharj, Saudi Arabia; 5 Department of Botanical and Environmental Sciences, Guru Nanak Dev University, Amritsar, 143005 India; 6 The UWA Institute of Agriculture and School of Agriculture & Environment, The University of Western Australia, LB 5005, Perth, WA 6001 Australia

## Abstract

The present study tested the efficacy of 24-epibrassinolide (EBL) and calcium (Ca) for mediating salinity tolerance in tomato. Salinity stress affected the morphological parameters of tomato as well as leaf relative water content (LRWC), photosynthetic and accessory pigments, leaf gas exchange parameters, chlorophyll fluorescence and the uptake of essential macronutrients. The salt (NaCl) treatment induced oxidative stress in the form of increased Na^+^ ion concentration by 146%, electrolyte leakage (EL) by 61.11%, lipid peroxidation (MDA) 167% and hydrogen peroxide (H_2_O_2_) content by 175%. Salt stress also enhanced antioxidant enzyme activities including those in the ascorbate–glutathione cycle. Plants treated with EBL or Ca after salt exposure mitigated the ill effects of salt stress, including oxidative stress, by reducing the uptake of Na^+^ ions by 52%. The combined dose of EBL + Ca reversed the salt-induced changes through an elevated pool of enzymes in the ascorbate–glutathione cycle, other antioxidants (superoxide dismutase, catalase), and osmoprotectants (proline, glycine betaine). Exogenously applied EBL and Ca help to optimize mineral nutrient status and enable tomato plants to tolerate salt toxicity. The ability of tomato plants to tolerate salt stress when supplemented with EBL and Ca was attributed to modifications to enzymatic and non-enzymatic antioxidants, osmolytes and metabolites.

## Introduction

Worldwide, the availability of agricultural land is shrinking gradually due to non-regulated and non-judicious agricultural practices, the rapid rise in industrialization, urbanization, and biotic and abiotic environmental pressures^1^. Of the various abiotic pressures, salinity is spreading across the globe due to salt water intrusion as a result of sea level rises in coastal areas, extensive irrigation practices in arid regions, and large-scale soil erosion^2^. It is estimated that, globally, approximately 7% of the total land area and 20% (~45 million ha) of the gross cultivable area are affected by the presence of high salt concentrations^3,4^. Salinity reduces crop yields worth billions of dollars every year^5^ and thus is a major abiotic constraint to crop yield and sustainable agricultural productivity^6^. Salinity causes ionic, oxidative and osmotic stress which retards plant growth and development^7–11^. Prolonged and high salt concentrations cause oxidative stress that generates reactive oxygen species (ROS), which oxidize biomolecules such as nucleic acids (DNA/RNA), proteins, lipid bilayer membranes, and enzyme inhibitors^12–16^. However, plants are equipped with defense mechanisms such as enzymatic and non-enzymatic antioxidants, osmoprotectants including proline and glycine betaine (GB), and enzymes in the ascorbate–glutathione (AsA–GSH) cycle *viz*. monodehydroascorbate reductase (MDHAR), ascorbate peroxidase (APX), dehydroascorbate reductase (DHAR) and glutathione reductase (GR), which neutralize the effects of salt-generated ROS^8,16,17^. Plant biologists are looking for alternatives to enhance crop production under increased salt stress. One strategy to combat salt stress is external supplementation of phytohormones.

Brassinosteroids (BRs) belong to a class of polyhydroxylated steroid phytohormones that are implicated in the resistance to a broad spectrum of environmental cues such as heavy metals, drought, pesticides and salinity^18–20^, Exogenous application of the natural BR, 24-epibrassinolide (EBR), has enhanced growth, pigment constituents, photosynthetic attributes, osmolytes and antioxidant enzyme activity in crop plants under abiotic stresses^20–23^. Foliar application of EBR to plants controls a range of biochemical and physiological responses to salt stress^24,25^.

Calcium (Ca) is a principal macronutrient in plants and is a ubiquitous secondary messenger in plant signaling. Ca is involved in the regulation of plant growth and development and the enhancement of abiotic stress tolerance^7,26–28^. It is well known that Ca plays a crucial role in controlling the structure, signaling and function of membranes by making bonds with the phospholipid bilayer, thus stabilizing and promoting the structural integrity of membrane organelles in plants exposed to stress environments^26,29^. Our earlier study showed that Ca application alleviates heavy metal toxicity in chickpea (*Cicer arietinum*) and Indian mustard (*Brassica juncea*) plants using various physiological and biochemical characteristics^26,27^. Khan, *et al*.^7^ showed that a combined treatment of Ca and gibberellin alleviated the effects of salt stress in linseed (*Linum usitatissimum*) plants better than 150 mM NaCl treatment. Al-Whaibi, *et al*.^30^ identified a coordinated role of salicylic acid and Ca that protects wheat (*Triticum aestivum*) plants from salinity stress. Rahman, *et al*.^31^ discovered that Ca supplementation to salt-stressed rice (*Oryza sativa*) seedlings minimized the impact of salt stress by improving ion homeostasis, and the antioxidant defense and glyoxalase systems. The synergistic role of EBR and Ca before and after seed sowing in the regulation of salinity-induced stress in tomato plants has not been reported. We evaluated the role of EBR and Ca on oxidative stress, antioxidant enzymes, and osmolytes in tomato seedlings. The study showed that EBR and Ca alleviated NaCl toxicity through modifications to antioxidant enzymes, osmolytes, the ascorbate–glutathione cycle and secondary metabolites.

## Materials and Methods

### Plant material and experimental setup

Healthy, uniform seeds of tomato (*Solanum lycopersicon* cv. K-21) were surface sterilized for 5 minutes with 5% NaOCl and then washed thoroughly with double-distilled water. The seeds were primed with 24-epibrassinolide (10^−7^ M) for 8 h and then sown in earthen pots containing a 3:1 mixture of perlite and sand. After germination, plants were thinned to three seedlings per pot. The pots containing seedlings were watered with full strength Hoagland’s nutrient solution (200 mL) every alternate day for 10 days. The salt stress (150 mM NaCl) treatment started 14 days after sowing by applying a modified Hoagland solution every week until day 40. After 14 days of salt stress, calcium (Ca)-in the form of calcium chloride (CaCl_2_; 50 mM)-was sprayed to plant foliage every alternate day until day 40. Control plants were provided with Hoagland’s nutrient solution only. The experimental pots were positioned in a complete randomized block design in a greenhouse with average day/night temperatures of 26 ± 2 °C, relative humidity of 70–75%, and an average photoperiod of 18 h light/6 h dark. The plants were harvested 40 days after sowing. Data presented in the manuscript is average data from three independent experiments, each treatment was replicated five times. Biochemical parameters and antioxidant enzyme activity were determined in the topmost fully grown young leaves.

### Determination of growth and photosynthetic pigment parameters

Shoot and root length was determined by scale. Samples were oven dried at 70 °C for 24 h and then weighed.

Photosynthetic pigment contents were analyzed using the acetone extract method^32^. Acetone (80%) was used to extract fresh leaf tissues, and absorbance read at 480 nm, 645 nm, 663 nm with a spectrophotometer (Beckman 640D, USA).

Leaf gas exchange measurements *viz*. net photosynthetic rate (*Pn*), carbon dioxide assimilation rate (*A*), stomatal conductance (*gs*) and transpiration rate (*E*) were measured on fully expanded horizontal leaves in full and bright sunlight between 10:00 h and 12:00 h using IRGA (LCA-4 model Analytical development Company, Hoddesdon England).

Chlorophyll fluorescence parameters were recorded with a junior PAM chlorophyll fluorometer (H. Walz, Effeltrich, Germany) on fully expanded horizontal tomato leaves^33^.

### Estimation of leaf relative water content (LRWC)

The standard protocol of Yamasaki and Dillenburg^34^ was adopted to estimate LRWC. Twenty leaf discs were punched from the upper most leaves, and their initial (fresh) weight noted. The discs were kept in double-distilled water for 60 min to become turgid and then weighed. The leaves were then oven dried at 70 °C for 24 h and then weighed. LRWC was calculated using the following formula:

$${\rm{LRWC}}={\rm{Fresh}}\,{\rm{weight}}-{\rm{Dry}}\,{\rm{weight}}/{\rm{Turgid}}\,{\rm{weight}}-{\rm{Dry}}\,{\rm{weight}}\times 100$$

### Determination of proline and glycine betaine (GB) content

The acid ninhydrin method of Bates, *et al*.^35^ was used to estimate proline content. A 500 mg fresh leaf sample was homogenized in sulfosalicylic acid and then centrifuged at 12,000 *g* for 8 min. The pellet was discarded, and a 2 mL aliquot of the supernatant added to equal volumes of acid ninhydrin and glacial acetic acid at 100 °C for 60 min. The slurry was placed on ice, and toluene blue was used to extract proline from samples before measuring the absorbance at 520 nm with a spectrophotometer (Beckman 640D, USA). The Pro content was determined from a standard curve and expressed as µM proline g^−1^ FW.

Glycine betaine content was determined according to the method of Grieve and Grattan^36^. Dry leaf material (500 mg) was extracted with 20 mL of double-distilled water after shaking at room temperature for 24 h. To the filtered extract, 2 N sulfuric acid was added. A 0.5 mL aliquot was reacted with 0.2 mL cold potassium iodide and centrifuged at 10000 *g* for 15 min. The supernatant was treated with 1,2-dichloroethane to dissolve the periodide-produced crystals. The reaction mixture was left undisturbed for 3 h before measuring the absorbance at 365 nm with a spectrophotometer (Beckman 640D, USA). The GB content was determined from a standard reference curve.

### Determination of oxidative stress biomarkers

Hydrogen peroxide (H_2_O_2_) was estimated using the method of Velikova, *et al*.^37^. Fresh leaf tissue (500 mg) was macerated with 0.1% trichloroacetic acid (TCA), and the homogenate centrifuged at 12,000 *g* for 8 min. The supernatant (0.5 mL) was mixed with 0.5 mL each of 100 mM potassium phosphate buffer (pH 7.0) and 1 M potassium iodide. The color was read at 390 nm with a spectrophotometer (Beckman 640D, USA). The H_2_O_2_ content was expressed as µM g^−1^ FW.

The standard protocol of Madhava Rao and Sresty^38^ was used to measure malondialdehyde (MDA) dependent content and to determine lipid peroxidation. Fresh leaf tissues were ground in 0.1% trichloroacetic acid (TCA) and centrifuged at 10,000 *g* for 5 min. Then, 4 mL of thiobarbituric acid (TBA) (prepared in 20% TBA) was added to 1 mL of supernatant and boiled at 100 °C for 30 min. The reaction mixture was terminated on an ice bath followed by centrifugation at 10,000 *g* for 10 min. The intensity of color formation was read at 530 and 600 nm with a spectrophotometer (Beckman 640D, USA).

The method of Dionisio-Sese and Tobita^39^ was followed to determine electrolyte leakage. Fresh leaf discs in test tubes containing 10 mL of double-distilled water were analyzed for their electrical conductivity (EC_0_). The sample tubes were boiled at 50 °C and 100 °C for 20 and 10 min, respectively, in a temperature-controlled dry block heater, and the respective electrical conductivities (EC_1_ and EC_2_) measured simultaneously. Electrolyte leakage was calculated using the following formula:

$${\rm{Electrolyte}}\,{\rm{leakage}}\,({\rm{EL}})=\frac{{\rm{EC}}1-{\rm{EC}}0}{{\rm{EC}}2-{\rm{EC}}0}\times 100$$

### Estimation of enzymatic antioxidant activities and the ascorbate-glutathione cycle Preparation of enzyme extract and assay

Fresh leaf material was collected and homogenized in a deep-freezer-cooled pestle and mortar in the presence of 1 mL of ice-cold 100 mM potassium phosphate buffer (pH 7.0) containing 1% of polyvinyl pyrrolidone. The slurry was centrifuged at 12,000 *g* for 30 min at 4 °C, and the resulting supernatant was used to determine different enzyme activities.

Superoxide dismutase activity (SOD, EC1.15.1.1) was measured using the nitroblue tetrazolium (NBT) reduction method of Dhindsa and Matowe^40^. The reaction mixture contained 1.5 ml of 100 mM phosphate buffer (pH 7.4), 0.2 ml of 10 mM methionine, 0.1 ml of 50 µM riboflavin, 100 µL extract of enzyme with equal amounts of 1 mM ethylenediaminetetraacetic acid (EDTA) and 70 µM nitro blue tetrazolium chloride (NBT). The mixture was kept under fluorescent tubes for 20 min, before measuring absorbance at 560 nm with a spectrophotometer (Beckman 640D, USA). One unit of SOD activity was defined as the amount of protein causing a 50% decrease of SOD-inhibitable NBT reduction and expressed in EU mg^−1^ protein.

Catalase (CAT: 1.11.1.6) activity was determined by monitoring the decomposition of H_2_O_2_ for 2 min at 240 nm with a spectrophotometer (Beckman 640D, USA)^41^. CAT activity was expressed in EU mg^–1^ protein.

Glutathione S-transferase (GST, 2.5.1.18) activity was estimated using the method of Hasanuzzaman and Fujita^42^. The reaction mixture was prepared by adding 100 mM Tris-HCl, buffer (pH 7.0), 1.0 mM GSH, 1 mM 1-chloro-2,4-dinitrobenzene (CDNB), and enzyme extract and the absorbance read at 340 nm with a spectrophotometer (Beckman 640 D, USA). GST activity was expressed in EU mg^−1^ protein.

Ascorbate peroxidase (APX, 1.11.1.11) activity was assayed according to Nakano and Asada^43^. The solution mixture containing potassium phosphate buffer (pH 7.0), ascorbate, H_2_O_2_, EDTA, and enzyme extract was prepared, and the H_2_O_2_-mediated oxidation of ascorbate was read at 290 nm for 2 min with a spectrophotometer (Beckman 640D, USA). APX activity was expressed in EU mg^−1^ protein.

Glutathione reductase (GR, 1.6.4.2) activity was measured according to the method of Foster and Hess^44^. The reaction mixture contained 100 mM potassium phosphate buffer (pH 7.0), 1 mM EDTA, 500 µM GSSG, 150 µM NADPH, and enzyme extract. The change in the intensity of absorbance color was read for 3 min at 340 nm with a spectrophotometer (Beckman 640D, USA). GR activity was expressed in EU mg^−1^ protein.

Monodehydroascorbate reductase (MDHAR, 1.6.5.4) activity was determined following the method of Miyake and Asada^45^. MDHAR activity was expressed in µmol NADPH oxidized (EU mg^−1^ protein).

Dehydroascorbate reductase (DHAR, 1.8.5.1) activity was assayed according to the standard protocol by Nakano and Asada^43^. The absorbance was recorded at 265 nm with a spectrophotometer (Beckman 640D, USA). DHAR activity was expressed in EU mg^−1^ protein.

Ascorbate (AsA) and glutathione (GSH) contents were measured according to the methods of Huang, *et al*.^46^ and Yu, *et al*.^47^. The glutathione content was determined by subtracting oxidized glutathione from total reduced glutathione.

### Determination of Na^+^ and other inorganic ions

Dry plant samples (0.5 g) were digested in a mixture of sulfuric acid and nitric acid (1/5, v/v) using a digestion assembly at 70 °C for 20 h. The solution was then treated with an acid mixture of HNO_3_/HClO_4_ (5/1, v/v) to make the solution colorless. The concentration of sodium and other ions was then determined with an atomic absorption spectrophotometer (Perkin-Elmer Analyst Model 300) and expressed in µM g^−1^ DW.

### Determination of flavonoid content

Fresh leaf material (500 mg) was ground and extracted in ethyl alcohol at 25 °C. The colorimetric method of Zhishen, *et al*.^48^ was used to estimate flavonoid content. For the calibration curve, catechin extract was used as a standard. The absorbance was recorded at 510 nm with a spectrophotometer (Beckman 640D, USA). Flavonoid content was expressed in mg catechin equivalents g^−1^ extract.

### Statistical analysis

The data were presented as the mean of five replicates ± standard error (SE). The data analysis was done with one-way analysis of variance (ANOVA) through the SPSS software (Version 17). The values at *P* ≤ 0.05 was treated as significant.

## Results

### Growth and biomass yield

Shoot length increased by 16.66%, 8.33% and 44.44% in the EBL, Ca and EBL + Ca treated control plants, respectively, relative to the control. Salt stress (150 mM) reduced shoot and root lengths by 59.89% and 39.60%, respectively, relative to the control plants (Table 1). NaCl-treated seedlings supplemented with EBL or Ca enhanced shoot length by 54.28% and 77.53%, respectively, compared to seedlings treated with NaCl alone. Root length increased by 16.98% with EBL and 21.31% with Ca in NaCl-treated seedlings, relative to NaCl alone. However, the combined dose of EBR + Ca to salt-stressed plants increased shoot and root lengths by 134.18% and 67.70%, respectively, compared to plants treated with NaCl alone.

**Table 1:** Pretreated seeds with EBL (10^−7^ M) and foliar application of Ca (50 mM) enhanced growth, biomass yield, total chlorophyll and carotenoids in tomato seedlings under NaCl stress.

Treatments	Shoot length (cm)	Root length (cm)	Shoot DW (g plant^−1^)	Root DW (g plant^−1^)	Total Chl (mg^−1^ FW)	Carotenoids (mg^−1^ FW)
0	25.31 ± 0.25c	11.11 ± 0.18c	0.36 ± 0.013b	0.23 ± 0.013d	1.38 ± 0.02c	0.51 ± 0.02f
EBL	27.95 ± 1.01b	12.01 ± 0.21b	0.42 ± 0.018bc	0.33 ± 0.025b	1.49 ± 0.03b	0.56 ± 0.01f
Ca	27.11 ± 1.07b	12.5 ± 0.23b	0.39 ± 0.016b	0.29 ± 0.018c	1.46 ± 0.02b	0.54 ± 0.02f
EBL + Ca	31.46 ± 1.09a	15.55 ± 0.53a	0.52 ± 0.048a	0.40 ± 0.029a	1.79 ± 0.07a	0.63 ± 0.02e
150 mM NaCl	10.15 ± 0.85g	6.71 ± 0.02f	0.12 ± 0.005g	0.09 ± 0.0008 h	0.79 ± 0.05f	0.78 ± 0.01d
150 mM NaCl + EBL	15.66 ± 0.41f	6.85 ± 0.03e	0.24 ± 0.011e	0.15 ± 0.007f	1.07 ± 0.02e	0.86 ± 0.01b
150 mM NaCl + Ca	18.02 ± 0.75e	8.14 ± 0.25d	0.21 ± 0.008f	0.12 ± 0.004g	1.04 ± 0.07e	0.82 ± 0.01c
150 mM NaCl + EBL + Ca	23.77 ± 0.51d	11.32 ± 0.12c	0.26 ± 0.009d	0.18 ± 0.009e	1.32 ± 0.01d	0.95 ± 0.02a

Data presented are the means ± SE (n = 5). Different letters next to numbers indicate significant differences at P ≤ 0.05.

Shoot dry weights decreased by 66.66% in NaCl-treated seedlings relative to control plants. Supplementing NaCl-treated seedlings with EBL or Ca enhanced shoot dry weight by 100.00% and 75.00%, respectively, compared to NaCl alone. The combined dose of EBR + Ca to NaCl-treated seedlings further increased shoot dry weight (by 116.66%) compared to NaCl alone (Table 1). NaCl decreased root dry weights by 60.86% over control plants, however, application of EBL, Ca and EBL + Ca to NaCl stressed plants enhanced root dry weight by 66.66%, 33.33% and 100.00% respectively over NaCl-alone treated plants.

### Photosynthetic pigments and leaf gas exchange

The total Chl content declined by 42.75% in NaCl-treated seedlings relative to control (Table 1). Supplementing NaCl-treated seedlings with EBR or Ca improved pigment contents by 35.44% and 31.64%, respectively, relative to NaCl alone. The combined dose of EBL + Ca to NaCl-treated seedlings further increased total Chl content (by 67.08%) compared to NaCl alone.

Carotenoid content increased by 52.94% in NaCl-treated seedlings relative to control plants. The application of EBL or Ca further increased this value by 10.25% and 5.12%, respectively, relative to NaCl alone. The combined dose of EBL + Ca to NaCl-treated seedlings increased the carotenoid content (by 21.79%) compared to NaCl alone (Table 1).

Salt stress reduced leaf gas exchange parameters—*Pn*, *A*, *gs* and *E*—by 20.31%, 48.02%, 81.32% and 67.45%, respectively, relative to the control. Pretreatment with EBR enhanced *Pn* by 29.80%, *A* by 32.11%, *gs* by 121.44% and *E* by 30.90% in NaCl-stressed seedlings, relative to NaCl alone, and Ca supplementation followed a similar ameliorating trend. The combined dose of EBL + Ca to NaCl-stressed plants enhanced *Pn*, *A*, *gs* and *E* by 90.51%, 65.10%, 301.28% and 74.54%, respectively, relative to NaCl alone (Table 2).

**Table 2:** Pretreated seeds with EBL (10^−7^ M) and foliar application of Ca (50 mM) enhanced gas exchange attributes in tomato seedlings under NaCl stress.

Treatments	Net photosynthesis rate *Pn* (mmol m^−2^ s^−1^)	CO_2_ assimilation *A* (mmol CO_2_ m^−2^ s^−1^)	Stomatal conductance *gs* (mmol CO_2_ m^−2^ s^−1^)	Transpiration rate *E* (mmol H_2_O m^−2^ s^−1^)
0	10.19 ± 0.66d	15.22 ± 0.01c	382 ± 5.66d	1.69 ± 0.041c
EBL	13.93 ± 0.37c	16.89 ± 0.07b	416 ± 2.54b	1.84 ± 0.005b
Ca	13.27 ± 0.81c	15.88 ± 0.03c	401 ± 1.32c	1.76 ± 0.023c
Ca + EBL	15.23 ± 0.03b	17.75 ± 0.02a	455 ± 6.81a	1.98 ± 0.021a
150 mM NaCl	8.12 ± 0.27f	7.91 ± 0.07g	71.35 ± 2.55g	0.55 ± 0.005g
150 mM NaCl + EBL	10.54 ± 0.69d	10.45 ± 0.05e	158 ± 3.71f	0.72 ± 0.009e
150 mM NaCl + Ca	9.89 ± 0.61e	8.98 ± 0.08f	156 ± 3.7f	0.67 ± 0.006f
150 mM NaCl + EBL + Ca	15.47 ± 0.07a	13.06 ± 0.01d	286.32 ± 4.25e	0.96 ± 0.078d

Data presented are means ± SE (n = 5). Different letters next to numbers indicate significant differences at P ≤ 0.05.

### Chlorophyll fluorescence parameters

Salt stress reduced the efficiency of PSII (Fv/Fm) by 34.61%, quantum yield of PSII (ΦPSII) by 23.43%, and photochemical efficiency (qp) by 27.47%, respectively, compared to control plants, but increased non-photochemical quenching (NPQ) by 41.79%. Pretreatment with EBR increased Fv/Fm by 43.13%, ΦPSII by 18.36% and qp by 13.63% but decreased NPQ by 18.94% in NaCl-stressed seedlings relative to NaCl alone. Supplementation with Ca enhanced Fv/Fm, ΦPSII, and qp by 35.29%, 10.20% and 9.09%, respectively, and decreased NPQ by 26.31% in NaCl-stressed seedlings relative to NaCl alone (Table 3). The combined dose of EBR + Ca enhanced Fv/Fm, ΦPSII, qp by 72.54%, 65.30%, and 33.33%, respectively, and decreased NPQ by 26.31% in NaCl-stressed seedlings relative to NaCl alone (Table 3).

**Table 3:** Pretreated seeds with EBL (10^−7^ M) and foliar application of Ca (50 mM) maintained chlorophyll fluorescence parameters in tomato seedlings under NaCl stress.

Treatments	Efficiency of PSII (Fv/Fm)	Quantum yield of PSII (FPSII)	Photochemical quenching (qp)	Non-photochemical quenching (NPQ)
0	0.78 ± 0.015d	0.64 ± 0.007c	0.91 ± 0.006b	0.67 ± 0.008d
EBL	0.89 ± 0.006b	0.67 ± 0.008c	0.94 ± 0.008a	0.48 ± 0.013e
Ca	0.83 ± 0.004c	0.64 ± 0.007c	0.93 ± 0.007a	0.41 ± 0.028e
Ca + EBL	0.93 ± 0.007a	0.71 ± 0.009b	0.96 ± 0.009a	0.35 ± 0.001f
150 mM NaCl	0.51 ± 0.005f	0.49 ± 0.004e	0.66 ± 0.001e	0.95 ± 0.006a
150 mM NaCl + EBL	0.73 ± 0.011d	0.58 ± 0.011d	0.75 ± 0.002d	0.77 ± 0.012b
150 mM NaCl + Ca	0.69 ± 0.003e	0.54 ± 0.009d	0.72 ± 0.001d	0.81 ± 0.012b
150 mM NaCl + EBL + Ca	0.88 ± 0.006b	0.81 ± 0.007a	0.88 ± 0.004c	0.7 ± 0.004c

Data presented are means ± SE (n = 5). Different letters next to numbers indicate significant differences at P ≤ 0.05.

### Leaf relative water content, proline and glycinebetaine

Salt stress reduced LRWC by 28.49% compared to control plants. The application of EBL or Ca to NaCl-stressed plants increased LRWC by 22.23% and 15.42%, respectively, relative to NaCl alone. The combined dose of EBL + Ca increased LRWC by 32.84% in NaCl-stressed seedlings relative to NaCl alone (Table 4).

**Table 4:** Pretreated seeds with EBL (10^−7^ M) and foliar application of Ca (50 mM) regulates LRWC, proline, glycinebetaine, H_2_O_2_, MDA content and electrolyte leakage in tomato seedlings under NaCl stress.

Treatments	LRWC (%)	Proline (mg g^−1^ FW)	GB (mg g^−1^ FW)	H_2_O_2_ (mmol g^−1^ FW)	MDA (mmol g^−1^ FW)	EL (%)
0	81.32 ± 0.85c	30.11 ± 1.72e	3.11 ± 0.035f	2.29 ± 0.02d	2.07 ± 0.08e	11.15 ± 0.25e
EBL	85.51 ± 0.81b	31.28 ± 1.75e	3.91 ± 0.045d	2.06 ± 0.01f	1.82 ± 0.04f	9.76 ± 0.85g
Ca	83.12 ± 0.38c	31.07 ± 1.75e	3.42 ± 0.038e	2.12 ± 0.01e	1.85 ± 0.02f	10.55 ± 0.04f
EBL + Ca	92.15 ± 1.7a	31.5 ± 1.76e	3.83 ± 0.041d	2 ± 0.03f	1.78 ± 0.01g	9.34 ± 0.09g
150 mM NaCl	58.15 ± 1.06f	105 ± 1.26d	4.85 ± 0.166c	6.29 ± 0.31a	5.52 ± 0.06a	61.11 ± 0.12a
150 mM NaCl + EBL	71.08 ± 1.21e	137 ± 0.84b	6.85 ± 0.175b	3.26 ± 0.15c	3.15 ± 0.03c	48.71 ± 0.45c
150 mM NaCl + Ca	67.12 ± 2.13e	133 ± 1.07c	6.59 ± 0.17b	3.89 ± 0.13b	3.56 ± 0.01b	53.12 ± 0.98b
150 mM NaCl + EBL + Ca	77.25 ± 0.82d	149 ± 1.88a	7.69 ± 0.186a	2.15 ± 0.04e	2.27 ± 0.06d	29.66 ± 0.49d

Data presented are means ± SE (n = 5). Different letters next to numbers indicate significant differences at P ≤ 0.05.

Salt stress increased the proline and GB contents by 3.48- and 1.55-fold relative to the control plants (Table 4). Pretreatment with EBL further enhanced the proline content by 1.30-fold and GB content by 1.41-fold compared to NaCl alone. Foliar supplementation with Ca further enhanced the proline content by 1.26-fold and GB content by 1.35-fold relative to NaCl alone. The EBL + Ca treatment further enhanced the proline content by 1.41-fold and GB content by 1.58-fold compared to NaCl alone.

### Hydrogen peroxide, malondialdehyde content and electrolyte leakage

Salt stress increased the production of H_2_O_2_, MDA, and EL by 174.67%, 166.66% and 448% relative to the control plants (Table 4). However, pretreatment with EBR decreased the production of H_2_O_2_ by 48.17%, MDA by 42.93% and EL by 20.29% relative to NaCl alone. Similarly, foliar supplementation with Ca reduced the production of H_2_O_2_ by 38.15%, MDA by 35.50% and EL by 13.07% relative to NaCl alone. The combined dose of EBR + Ca was more effective at reducing stress in NaCl-stressed seedlings than the individual applications of EBL or Ca, with reductions of 65.85%, 58.87% and 51.47% in H_2_O_2_, MDA, and EL production, respectively, compared to NaCl alone.

### Activity of antioxidant enzymes and enzymes of ascorbate–glutathione cycle

**Figure 1:**
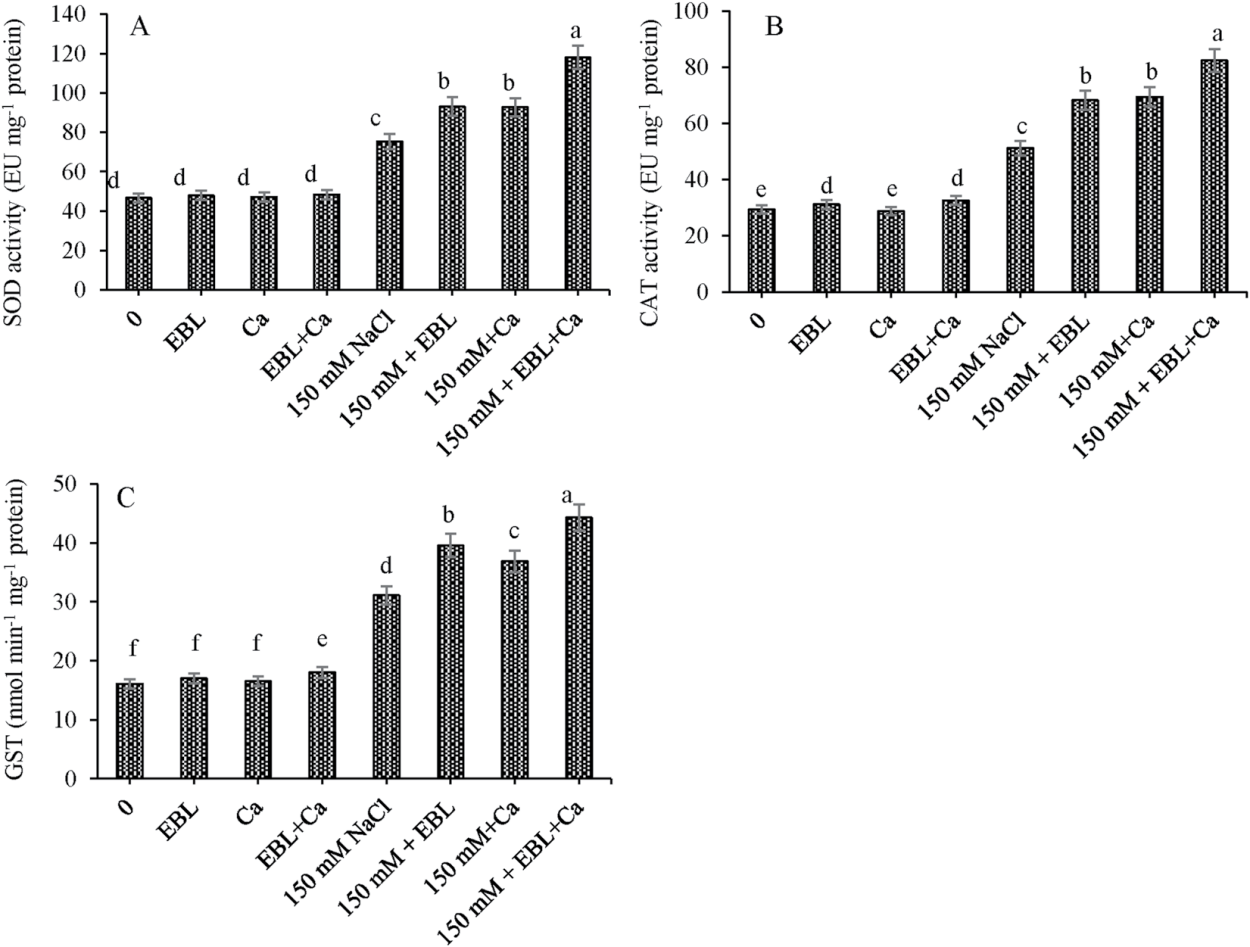
Pretreatment of seeds with EBL (10^−7^ M) and foliar application of Ca (50 mM) enhanced the activity of (**A**) SOD (**B**) CAT, and (**C**) GST in tomato seedlings under NaCl stress. Data presented are means ± SE (n = 5). Different letters indicate significant differences at P ≤ 0.05.

Salt stress elevated SOD and CAT activities by 61.43 and 74.34%, respectively, relative to control seedlings. Pretreatment with EBR further increased SOD activity by 23.63% and CAT activity by 33.32% in NaCl-stressed seedlings compared to NaCl alone. Foliar supplementation with Ca increased SOD and CAT activities by 23.18% and 35.66%, respectively, relative to NaCl alone. The combined dose of EBL + Ca increased SOD activity by 56.68% and CAT activity by 60.97% relative to NaCl alone (Fig. 1A,B).

Salt stress increased GST content by 93.76% relative to the control seedlings, which further increased by 27.16%, 18.54% and 42.46% with the EBL, Ca and EBL + Ca treatments, respectively, relative to NaCl alone (Fig. 1C).

**Figure 2:**
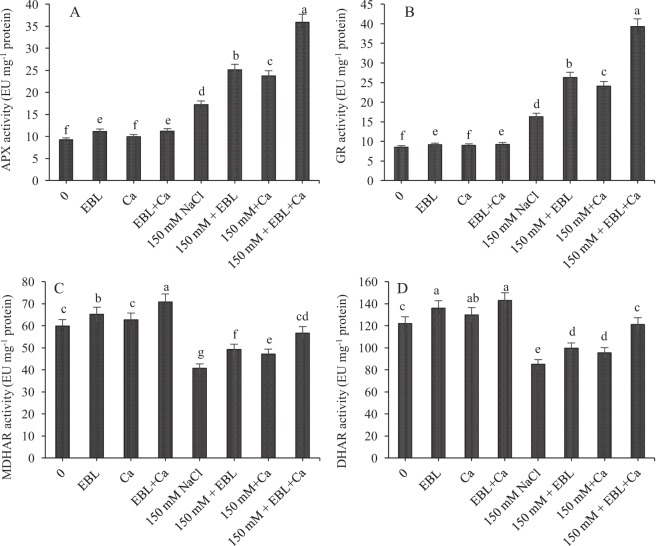
Pretreatment of seeds with EBL (10^−7^ M) and foliar application of Ca (50 mM) enhanced the activity of (**A**) APX (**B**) GR (**C**) MDHAR and (**D**) DHAR in tomato seedlings under NaCl stress. Data presented are means ± SE (n = 5). Different letters indicate significant differences at P ≤ 0.05.

The activity of the four enzymes in the AsA–GSH pathway—APX, GR, MDHAR, and DHAR—differed in their response to salt stress (Fig. 2A–D). Salt stress increased APX and GR activities by 86.05% and 91.89%, respectively, compared to control seedlings, but MDHAR and DHAR activities decreased by 32.00% and 30.19%, respectively (Fig. 1B). Pretreatment with EBL further enhanced the activity of APX by 46.13%, GR by 61.05%, MDHAR by 21.00% and DHAR by 16.90% in NaCl-stressed seedlings relative to NaCl alone. Supplementation of Ca to NaCl-stressed seedlings increased the activity of APX by 37.67%, GR by 47.51%, MDHAR by 39.35% and DHAR by 42.39% relative to NaCl alone. The combined dose of EBL + Ca further enhanced APX, GR, MDHAR and DHAR activities by 108.54%, 140.47%, 39.35% and 42.39%, respectively, in NaCl-stressed seedlings relative to NaCl alone.

### Non-enzymatic antioxidants

**Figure 3:**
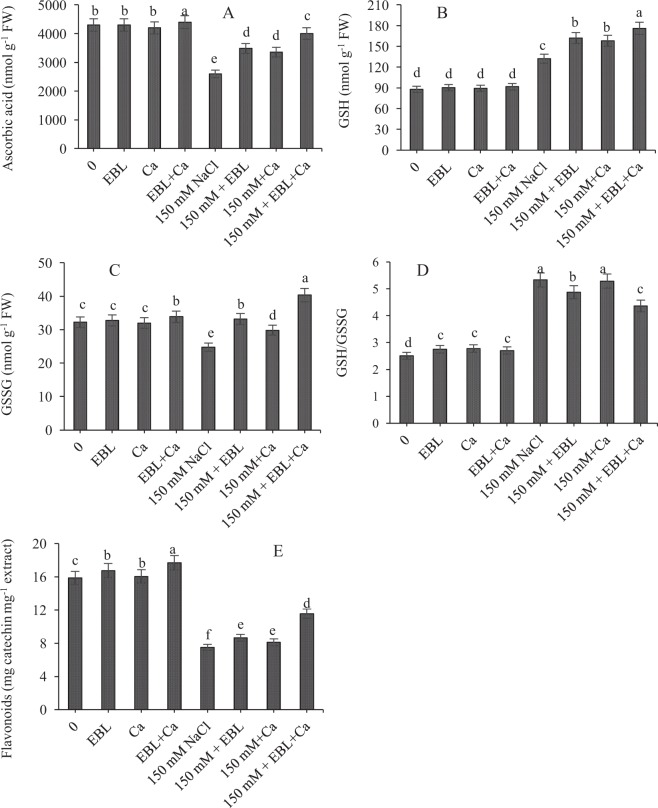
Pretreatment of seeds with EBL (10^−7^ M) and foliar application of Ca (50 mM) maintained (**A**) AsA, (**B**) GSH, (**C**) GSSG, (**D**) GSH/GSSG ratio and (**E**) flavonoid content in tomato seedlings under NaCl stress. Data presented are means ± SE (n = 5). Different letters indicate significant differences at P ≤ 0.05.

Salt stress reduced the ascorbate (AsA) content by 39.53% compared to the control. However, AsA content increased by 33.84% with EBL pretreatment and 28.84% with Ca supplementation in NaCl-stressed seedlings compared to NaCl alone. The combined dose of EBL + Ca further enhanced AsA content by 53.84% in NaCl-stressed seedlings relative to NaCl alone (Fig. 3A).

Salt stress increased GSH production by 50.01% relative to control seedlings. GSH production further increased by 22.72% with EBL pretreatment and 19.96% with Ca supplementation in NaCl-stressed seedlings relative to NaCl alone. The combined dose of EBR + Ca in NaCl-stressed seedlings further increased GSH production by 33.33% relative to NaCl alone (Fig. 3B).

Salt stress decreased GSSG content by 23.27% relative to control seedlings. However, EBL, Ca and EBL + Ca supplementation increased GSSG content in NaCl-stressed seedlings by 34.10%, 20.63% and 63.22%, respectively, relative to NaCl alone (Fig. 3C).

Salt stress increased the redox state (ratio of GSH/GSSG) by 112.35% relative to the control seedlings. The ratio of GSH/GSSG in NaCl-stressed seedlings in the EBR, Ca and EBR + Ca treatments decreased by 94.42%, 0.75% and 18.19% relative to NaCl alone (Fig. 3D).

### Flavonoids

Salt stress reduced flavonoid content by 52.64% relative to control seedlings. However, supplementation with EBL, Ca and EBL + Ca to NaCl-stressed seedlings further enhanced flavonoid content by 15.31%, 8.12% and 56.66%, respectively, relative to NaCl alone (Fig. 3E).

### Nutrient elements

Salt stress increased the Na^+^ ion concentration by 146.29% and Na^+^/K^+^ ratio by 773% and decreased K^+^ and Ca^+^ ion concentrations by 71.80% and 44.54%, respectively, relative to the control. EBL supplementation reduced the Na^+^ concentration by 32.67% and Na^+^/K^+^ ratio by 75.23%, and increased the K^+^ and Ca^2+^ concentrations by 107.75% and 74.91% in the NaCl-stressed seedlings relative to NaCl alone. Supplementation with Ca followed a similar trend. However, the combined dose of EBL + Ca to NaCl-stressed seedlings reduced the Na^+^ concentration and Na/K ratio by 51.84% and 84.57%, respectively, and increased the K^+^ and Ca^2+^ concentrations by 211.40% and 141.01%, respectively, relative to NaCl alone (Table 5).

**Table 5:** Pretreated seeds with EBL (10^−7^ M) and foliar application of Ca (50 mM) maintained Na^+^, K^+^, Na/K and Ca^2+^ uptake in tomato seedlings under NaCl stress.

Treatments	Na^+^	K^+^	Na/K ratio	Ca^2+^
0	15.27 ± 0.97e	31.11 ± 0.52b	0.49 ± 0.006e	5.32 ± 0.042e
EBL	14.11 ± 0.71e	34.27 ± 0.66a	0.41 ± 0.004f	8.09 ± 0.067b
Ca	12.21 ± 0.93f	33.21 ± 0.6a	0.36 ± 0.003g	7.87 ± 0.033c
EBL + Ca	11.06 ± 0.87g	35.25 ± 0.62a	0.31 ± 0.001 h	9.25 ± 0.088a
150 mM NaCl	37.61 ± 1.55a	8.77 ± 0.75e	4.28 ± 0.031a	2.95 ± 0.052g
150 mM NaCl + EBL	25.32 ± 0.23c	18.22 ± 0.25d	1.06 ± 0.008c	5.16 ± 0.037e
150 mM NaCl + Ca	29.32 ± 1.27b	17.19 ± 0.53d	1.7 ± 0.003b	4.91 ± 0.033f
150 mM NaCl + EBL + Ca	18.11 ± 0.41d	27.31 ± 0.25c	0.66 ± 0.005d	7.11 ± 0.056d

Data presented are means ± SE (n = 5). Different letters next to numbers indicate significant differences at P ≤ 0.05.

## Discussion

### Growth and biomass yield

Salt stress reduced the growth and biomass yield (shoot dry weight) of tomato seedlings, and the findings are consistent with our earlier results in chickpea^49^. Salt stress also reduces plant growth and biomass yield in tomato^50^, wheat^51^ and rice^52^ etc. and possibly due to the inhibition of nutrient uptake to plants. Supplementation with EBR and Ca, individually or combined, enhanced the recovery of plant growth related parameters. Supplementation with EBR has increased growth and yield parameters in various plants under salt stress, including pepper (*Capsicum annuum*)^53^, pea (*Pisum sativum*)^54^ and Indian mustard^23^. Similarly, Ca supplementation has alleviated the inhibitory growth effects of salt stress in species such as linseed^7^, rice^31^, pistachio^55^ and Indian mustard^56^. EBL increased growth in *Vigna radiata* under salt stress by enhancing the photosynthetic rate and carbonic anhydrase activity^57^. EBR plays a role in H^+^-ATPase activation^58^ that is directly responsible for the activation of cell wall loosening enzymes and therefore improving growth. According to Haubrick and Assmann^59^, brassinosteroids are involved in cell elongation and germination due to their interaction with other growth regulators. The growth promoting and ameliorating ability of EBR may be due to the modulation of cellulose biosynthesis and enhanced rates of cell division and cell elongation which ultimately lead to enhanced plant growth^60^. Calcium supplementation reduced the effects of cadmium stress on growth and biomass yield in Indian mustard, which was attributed to the uptake of mineral elements by Ca^26^.

### Pigments and leaf gas exchange

The photosynthetic potential of plants reflects their overall performance, which is manifested by various biomass and growth parameters. Salt stress negatively impacts chlorophyll content, leading to its impaired biosynthesis and accelerated pigment degradation^61^. Salt-driven stress also destabilizes pigments associated with the chlorophyll protein complex and reduces the amount of photosynthetic pigments by accelerating the activity of chlorophyllase^1^. Plants supplemented with EBR or Ca increased their photosynthetic potential in the absence of salinity. However, combined effect of EBR + Ca showed maximum photosynthetic efficiency under both control as well as salt stress. Supplementation with EBL to NaCl-stressed plants enhanced chlorophyll synthesis, which is likely due to its role in regulating stress-causing agents^62^. According to Li, *et al*.^63^, enhanced expression of the BR biosynthetic pathway enhances the activity of Calvin cycle enzymes that boost photosynthesis. Under salt stress, EBR and Ca reduced the uptake of Na^+^ ions in tomato seedlings and increased the activity of enzymes associated with carbon reactions of photosynthesis. All these positive effects of EBR and Ca alleviated the inhibition of photosynthetic capacity under salt stress. These findings are consistent with those of Hayat, *et al*.^57^ in *V*. *radiata*, Ahmad, *et al*.^20^ in *C*. *arietinum*, Ahmad, *et al*.^26^ in *B*. *juncea* and Rahman, *et al*.^31^ in *O*. *sativa*. EBL supplementation can assist mineral uptake especially Mg^2+^ during mercury (Hg) stress^20^, which may explain the increase in chlorophyll content in tomato seedlings under salt stress as Mg^2+^, being the central element of the chlorophyll molecule, decreased under NaCl stress. Choudhary, *et al*.^64^ reported that EBL supplementation enhanced photosynthetic pigments in radish (*Raphanus sativus*) under chromium stress. Lechowski and Białczyk^65^ reported that Ca served as a second messenger for cytokinin action in boosting chlorophyll synthesis.

Carotenoids play a major role in the photosynthetic reaction center by regulating photo protection against auto-oxidation^66^. The enhanced biosynthesis of carotenoids during salt stress in the present study may be due to carotenoids acting as an antioxidant and assisting in oxidative stress management^27,49^. Increased carotenoid biosynthesis with EBR supplementation is also due to phytoene synthase (PSY), a key enzyme in its biosynthetic pathway^18^. Additionally, supplementation of EBR and Ca further increased the carotenoid content, which reduced the oxidative damage caused by salt toxicity; this is supported by studies on two varieties of pepper^53^, and chickpea^20^.

The reduction in leaf gas exchange parameters [CO_2_ assimilation rate (*A*), transpiration rate (*E*) and stomatal conductance (*g*_*s*_)] under NaCl stress in the present study agrees with similar studies on maize (*Zea mays*)^67^, cucumber (*Cucumis sativus*)^68^ and mung bean (*V*. *radiata*)^69^. Similarly, salinity stress reduced photosynthesis (*P*_*N*_) and *g*_*s*_ in mung bean^57^ and cucumber^22^. Salt-induced osmotic stress results in stomatal closure thereby slowing the transpiration rate. Stomatal closure also leads to a reduction in photosynthetic rate as the fixation of CO_2_ decreases. Salt-induced reductions in *P*_*N*_ have been attributed to protein dysfunction due to altered enzyme activities and negative feedback by reduced sink activity^70^. Supplementation with EBL enhanced gas exchange parameters in the present study and is supported by studies on salt-stressed watermelon (*Citrullus lanatus*)^71^ and water-stressed cowpea (*Vigna unguiculata*)^72^ and drought stressed capsicum (*C*. *annuum*)^73^. The activity of Rubisco enzyme was activated by brassinosteroids^74^ and may be due to an enhanced CO_2_ assimilatory rate^75^. BRs have also enhanced the expression of specific genes in *B*. *juncea* under pesticide stress^18^. Application of Ca enhanced gas exchange parameters in a perennial lawn species (*Zoysia japonica*) under drought stress^76^. Ca helps plants to maintain relative water contents and stomatal conductance^27^ thus preventing damage from cytoplasm dehydration^77^.

### Chlorophyll fluorescence

Chlorophyll fluorescence is a widely used alternative method to quantify tolerance and acclimation of plants to environmental extremes^78^. Salt stress decreased the Fv/Fm value in the present study, which is consistent with the findings in Indian mustard^23^. In our study, salt stress reduced ΦPSII and qP in tomato seedlings, which was also reported in salt-stressed eggplant (*Solanum melongena*)^79^ and water-stressed cowpea (*Vigna unguiculata*)^72^. Salt stress can affect PSII electron transport^80^ and their photoinhibition results in destruction in antenna pigments. Salt stress blocks electron transfer from the primary acceptor (plastoquinone, *Q*_A_) at the secondary (plastoquinone, *Q*_B_) at the acceptor side of PSII leading to a decline in the Fv/Fm ratio^81^. Supplementation with EBR reversed the negative effects of NaCl on the Fv/Fm ratio, ΦPSII, qP and NPQ in tomato seedlings, which is consistent with findings in Indian mustard^23^, cucumber^22,24^. Brassinosteroids maintained the Fv/Fm ratio, ΦPSII and qp in wheat^82^ and eggplant^79^ under NaCl stress and also reduced PSII photoinhibition in wheat^83^. In the present study, EBR supplementation to salt-stressed tomato seedlings reduced NPQ, which is similar to the findings in *Solanum melongena* (L.) plants^79^. Application of Ca enhanced the Fv/Fm ratio, ΦPSII, qP and reduced NPQ in the salt-stressed tomato seedlings in the present study. This positive role of Ca may be attributed to the mineral ion homeostasis and uptake of water under NaCl stress^31^. These results suggest that EBR and Ca protect the PSII from over-excitation and maintain the structural integrity of the thylakoid membrane.

### Leaf relative water content, proline and glycinebetaine

Salt stress significantly reduced LRWC in tomato seedlings in the present study, which is likely due to salt-induced constraints to the availability and uptake of water and injury to root system architecture^27,49^. Supplementation with EBR and Ca had a positive effect on LRWC under salt stress by reducing membrane injury and improving the water balance. A similar response has been observed with exogenous Ca application in linseed (*Linum usitatissimum*)^7^, Indian mustard (*B*. *juncea*^8^) and rice (*O*. *sativa*^31^) and EBR supplementation in ryegrass (*Lolium rigidum*^25^) and pepper (*C*. *annuum*^84^). Supplementation of 24-EBL to Cd-stressed common bean (*Phaseolus vulgaris*) enhanced proline content and LRWC^85^ thus efficient water uptake is related to EBL action. Calcium application also assists water and mineral uptake as it controls the transport of Na^+^ ions^27^.

To combat the negative effects of salt stress, plants trigger the production of osmolytic cytosolutes^86^. Proline and GB helped to alleviate salt stress in Indian mustard^87^, rice^14^, mung bean^13^, linseed^7^, mulberry^11^. Both of these osmolytes help in cell osmoregulation under salinity^13,49^. Proline shields the photosynthetic machinery and acts as a molecular chaperone, energy storage, and protects membranes and enzyme activity^88,89^. Proline can also reduce oxidative stress by neutralizing the effect of free radicals, bringing down the elevated levels of H_2_O_2_ and MDA, and increasing the activities of those enzymes associated with ROS scavenging^90^. GB detoxifies excess ROS, maintains proper functioning of photosynthetic machinery, and modulates gene activation related to stress^17,91^. GB also plays an adaptive role in controlling osmotic adjustment and protecting cellular and subcellular constituents, protecting machinery at the transcriptional and translational level, and as a molecular chaperone in the folding of enzymes to protect proteins from damage induced by various abiotic stresses^92^. GB prevents the inactivation of Rubisco and the oxygen-evolving complex of PSII^93^. Hence, in our study, proline and GB maintained their elevated levels in tomato seedlings during salt stress, and these levels increased further with supplementation of EBR and Ca. Epibrassinolide enhances the proline content in many plant species under stress^94,95^. Yusuf, *et al*.^96^ reported an increase in proline content and proline metabolism enzymes’ activities after application of epibrassinolide to salt-stressed wheat plants. Enhanced synthesis of proline and GB is related to the restoration of photosynthetic efficiency and photoassimilate production, plant growth and reduces salt mediated oxidative stress^7,14^. Foliar application of proline in *B*. *juncea* also mitigated the salt driven changes and overwhelmed the salt stress changes in growth, photosynthesis, and yield parameters^97^. Ca is an important signaling molecule and is involved in proline biosynthesis^98^. According to Yoo, *et al*.^99^, a calcium-signaling unit (calmodulin) activates the transcription factor MYB2 (myeloblastosis), which in turn activates many downstream genes including *pyrroline-5-carboxlyate synthetase* 1 (*P5CS1*). Parre, *et al*.^100^ reported overexpression of the *P5CS1* gene under salt stress in Arabidopsis by another Ca-signaling component, phospholipase “C”.

### Hydrogen peroxide, malondialdehyde content and electrolyte leakage

H_2_O_2_ and MDA production and EL are indicators of oxidative stress biomarkers under abiotic stress^14,20^. Tomato plants under salt stress produced more MDA and H_2_O_2_ than control plants, which consequently increased EL; these results are similar to those in wheat^101^, and chickpea^1,27,49^. Supplementation with EBR and Ca in salt-stressed tomato seedlings reduced these values indicating the efficacy of EBR and Ca to alleviate salt-induced oxidative stress. These findings agree with previous studies on *O*. *sativa*^21,31,102^, tomato^103^. Supplementation with EBR reduced salt-driven oxidative stress in tomato seedlings more so in combination with Ca than an individual application. EBL supplementation enhanced membrane stability in iron-deficient peanuts^104^, and decreased the production of H_2_O_2_ in chickpea seedlings under mercury stress^20^ which in turn reduced lipid peroxidation and electrolyte leakage. Thus, EBL can be directly related to membrane protection under salinity stress. Ca bonds to the phospholipid bilayer of cellular membranes thus stabilizing the lipid bilayer and providing structural integrity during stress^105^. Similar findings related to the protective role of Ca and EBR during saline and other stress environments have been substantiated in Indian mustard^56^, wheat^96^, mung bean^106^, chickpea^27^, and tomato^107^. Therefore, a combined dose of EBR + Ca could serve as an effective practice to reduce salt-induced oxidative stress.

### Antioxidants and the ascorbate–glutathione cycle

Plants have evolved efficient resistance mechanisms, such as enzymatic and non-enzymatic antioxidants, which regulate oxidation reactions and protect plant cells from oxidative damage by scavenging ROS^108^. SOD is considered the first line of defense against abiotic-induced oxidative stress. It catalyzes the dismutation reaction of the superoxide anion (${{\rm{O}}}_{2}^{\cdot -}$) into oxygen (O_2_) and H_2_O_2_ and this oxidant H_2_O_2_ is reduced by APX, CAT and GPX to water^109^. In the present study, both APX and CAT activity increased in tomato seedlings in response to salt stress, which is a similar response in other species like *O*. *sativa*^110^, *C*. *arietinum*^1^, *B*. *juncea*^3,4,26^.

CAT has a high turnover rate, but with lower affinity for H_2_O_2_ than APX. It is generally accepted that CAT involves the removal of overproduced H_2_O_2_ during oxidative stress^27,49^. NaCl enhanced SOD, CAT, APX and GR activities in chickpea^1^, tomato^111^ and wheat^112^, Acacia^113^. Application of EBR further enhanced antioxidant enzyme activity in the present study, which is consistent with the findings in cucumber^22,24^, mung bean^57^, wheat^114^ under NaCl stress. The H_2_O_2_ produced is detoxified by APX into H_2_O using AsA as the substrate. The increased GR activity after salt stress provides GSH which reduces DHAR to dehydroascorbate (DHA) to AsA via the AsA–GSH cycle. GSH is oxidized to GSSG and subsequently recycled by GR, thus, the ratio of GSH/GSSG is important for sustaining the cell redox state^115^. GR activity is enhanced by EBL supplementation and protects the photosynthetic machinery from superoxide radicals by maintaining the NADP^+^ concentration for electron transport^57^. According to Yuan, *et al*.^116^, EBL enhances GR activity to maintain the GSH/GSSG ratio for normal cell functioning. Upregulation of GR activity with EBL application protects the photosynthetic apparatus from the toxic effects of superoxide radicals by maintaining optimal concentrations of NADP^+^ for electron transport^57^.

Ascorbate and glutathione are powerful antioxidants^117^ and they serve as redox buffering agents and prevent oxidation of the plasma membrane^118^. Glutathione and ascorbate can donate electrons for key enzymes such as APX and GPX^119^. Other enzymes in the ascorbate–glutathione cycle (MDHAR, DHAR, GR) also play a role in the management of oxidative stress tolerance^120,121^. In the present study, the GSH/GSSG ratio, DHAR and MDHAR activities, and AsA content in salt-stressed tomato seedlings declined, but EBR and Ca restored and upregulated the enzyme activities. EBR enhanced the synthesis of GSH and AsA through the activation of MDHAR and DHAR enzymes thus providing a maximum supply of AsA to APX and GSSG to GR^103^. Increased MDHAR and DHAR activities with EBL supplementation have also been reported in *B*. *juncea*^122^, *Acacia gerrardii*^113^ and *Solanum lycopersicum*^123^. Salt-stressed tomato seedlings supplemented with Ca and EBR had much higher MDHAR and DHAR activities than the controls, which suggests that the radicals produced by APX during the H_2_O_2_ reduction reaction to water were transferred immediately back into AsA via MDHAR or through spontaneous disproportion processes^123^.

GSH plays a significant role in maintaining the GSH/GSSG ratio in the conversion of GSSG to GSH. The combined application of EBR + Ca increased GSH production thereby converting more GSSG to its reduced form and creating a reduced redox homeostatic environment. Glutathione S-transferase is an enzyme which enhances plants’ survival under salt stress conditions^124^. In the present study GST increased in all the treatments viz, NaCl, NaCl + EBR, NaCl + EBR + Ca in tomato seedlings, which indicates a promising role of thiols in the detoxification of salinity stress. EBR enhanced the activity of GST and suppressed lipid peroxy-radicals in tomato plants under hydrocarbon stress^123^. Thus it may be concluded that from above discussion that EBR and Ca modulated the AsA-GSH cycle to a redox state in presence of salt stress which plays a profound role in imparting salt stress tolerance to tomato. The whole summary of antioxidants has been apprehensively depicted in.

### Flavonoids

Plants that accumulate higher flavonoid content have greater salt tolerance than plants with low flavonoid-accumulating capacity^125^. Flavonoid content increases in many plants subjected to salt stress, and being electron donating agents, they have antioxidative properties to scavenge ROS^126^. Salt stress enhanced flavonoid content in black nightshade (*Solanum nigrum*^127^) and common bean (*Phaseolus vulgaris*)^128^. Oxidative stress imparts pressure on the flavonoid pathway to enhance flavonoid synthesis^129^. Abiotic stress frequently enhances flavonoid content to protect plants from osmotic and oxidative stress^27^. Flavonoids inhibit the lipoxygenase enzyme that is responsible for the conversion of polyunsaturated fatty acids to oxygen-containing derivatives^130^. Accumulation of these flavonoids helps to decrease lipid peroxidation and strengthen membrane protection^131^. Application of brassinosteroids enhanced the flavonoid content in heart-leaved moonseed (*Tinospora cordifolia*) with the highest concentration reported in leaves^132^, tomato^123^ and tea plants^133^, Similarly, Ca supplementation enhanced the flavonoid content in chickpea^27^. Enhanced flavonoid content in Ca-supplied NaCl-stressed seedlings is attributed to (i) restricted uptake of Na^+^ ions by Ca, and (ii) induction of gene expression for polyphenol biosynthesis^76^. Thus, EBR and Ca synergistically increase the ROS scavenging capacity which may correspond to the increase in flavonoid content under salt stress in the present study.

### Ion accumulation

Under saline conditions, Na^+^ ions surround the rhizosphere ready to enter the root; this causes a large electrochemical gradient of ions, which results in an influx of Na^+^ ions via membrane-located channels and transporters on the plasma membrane^134,135^. The antagonistic effect that persists between Na^+^ and essential minerals ions, such as K^+^ and Ca^2+^, at their site of uptake results in an ion imbalance by altering Ca^2+^ and the ratios of Na^+^/K^+^ under salt stress. In our study, we reported an influx of excess Na^+^ ions which elevated their endogenous concentration to trigger K^+^ efflux, reflected in the low K^+^ content, resulting in disturbed ion homeostasis which may displace Ca^2+^ by Na^+^. The salt ion influx in to the cell and mineral ion leakage from the cell may also lead to higher ROS accumulation. However, both EBR and Ca maintained mineral ion homeostasis by increasing Ca^2+^ and K^+^ concentrations and decreasing the Na^+^/K^+^ ratio under salt stress and control conditions, thereby conferring salt tolerance by regulating ROS production. Ca and K are required by plants for various enzymatic activities, and a deficiency of these elements under NaCl stress will inhibit protein synthesis and reduce growth^136^. Supplementation with EBR enhanced Ca^2+^ and K^+^ concentrations and the K^+^/Na^+^ ratio in wheat under salt stress^136^. Supplementation of Ca^2+^ externally enhanced the uptake of mineral elements thus improves photosynthesis^137^, and at the same time hampers Na^+^ uptake.

### Conclusion and future prospectus

The present study revealed that salt stress has a negative impact on growth and photosynthetic pigments in tomato seedlings. It also increases the production of ROS which causes lipid peroxidation and electrolyte leakage. Pretreatment of seeds with EBL and supplementation of Ca enhanced growth and the pigment system and decreased ROS accumulation through the scavenging activities of antioxidants. EBL and Ca reversed the negative impact of NaCl stress through the modulation of physiological attributes, biochemical parameters, and enzymatic and non-enzymatic activities of antioxidants. Our results demonstrate that supplementation with EBR and Ca to restrain salt stress could pave the way forward to boost salt-stress tolerance in salt-challenged fields which is vital to future crop productivity.
